# An exploration of trolling behaviours in Australian adolescents: An online survey

**DOI:** 10.1371/journal.pone.0284378

**Published:** 2023-04-12

**Authors:** Jessica Z. Marrington, Evita March, Sarah Murray, Carla Jeffries, Tanya Machin, Sonja March

**Affiliations:** 1 School of Psychology and Wellbeing and Centre for Health Research, University of Southern Queensland, Ipswich, Queensland, Australia; 2 Institute of Health and Wellbeing and Health Innovation and Transformation Centre, Federation University Australia, Berwick, Australia; University of Padova, ITALY

## Abstract

To understand why people “troll” (i.e., engage in disruptive online behaviour intended to provoke and distress for one’s own amusement), researchers have explored a range of individual differences. These studies have primarily been conducted in adult samples, despite adolescents being a particularly vulnerable group with regards to both being trolled and trolling others. In this study we aimed to (1) explore Australian adolescents’ experiences of trolling, and (2) replicate adult research that has constructed a psychological profile of the Internet troll by examining the utility of personality traits (psychopathy and sadism), self-esteem, empathy (cognitive and affective), and social rewards (negative social potency) to predict adolescents’ trolling behaviours. A sample of 157 Australian adolescents (40.8% male, 58% female, 0.6% non-binary) aged 13–18 years (*M* = 15.58, *SD* = 1.71) completed the Global Assessment of Internet Trolling-Revised, Adolescent Measure of Empathy and Sympathy, Rosenberg Self-Esteem Scale, Youth Psychopathy Traits Inventory-Short Version, Social Rewards Questionnaire, Short Sadistic Impulse Scale, and a series of questions related to the experience of trolling. Results showed in the past year, 24.2% of Australian adolescents reported being trolled and 13.4% reported having trolled others. Gender, psychopathy, sadism, self-esteem, cognitive empathy, affective empathy, and “negative social potency” (i.e., enjoyment of antisocial rewards) combined, explained 30.7% of variance in adolescents’ trolling behaviours (*p* < .001). When accounting for shared variance, gender (male), high psychopathy, and high negative social potency were significant predictors of trolling, aligning with findings of adult samples. Contrary to adult samples, sadism was not a unique predictor of adolescents’ trolling. For adolescents, the variance in trolling explained by sadism was nonsignificant when controlling for negative social potency. These similarities, and differences, in predictors of trolling across adult and adolescent samples may play a critical role in the development of targeted interventions to prevent or manage trolling.

## Introduction

Social media use has a range of benefits for adolescents, including opportunities for learning and entertainment [1], peer connections [2], and promoting positive mental health [3]. Despite these benefits, there is also opportunity for adolescents to experience antisocial online behaviour, such as cyberbullying [4] and sexual exploitation [5]. Although some antisocial online behaviours, such as cyberbullying (i.e., repeated online aggression and harassment [6]), have been extensively explored in adolescent populations, comparatively little research attention has been paid to Internet trolling (i.e., “trolling”). Trolling, an online antisocial behaviour, is qualitatively distinct from cyberbullying and appears to have different motivators, at least in adults [7]. Despite recommendations that researchers pay particular attention to adolescents and trolling [8], to date, there remains a paucity in studies that have explicitly documented the rate of trolling in a group of adolescents, and explored whether the psychological profile of the adult Internet “troll” can be replicated for adolescents. In the current study, we address this gap in the extant literature of adolescents and online antisocial behaviour. Based on recommendations of previous researchers [9], we propose that by understanding predictors of trolling, we take informed steps towards evidence-based management and intervention of the potentially harmful online behaviour, in a population that may be particularly vulnerable to being trolled and trolling others [8].

### Internet trolling: Definition and impact

Broadly, trolling has been adopted as an umbrella term to describe a range of antisocial deviant online behaviours [10]. In the current study, we follow previous empirical definitions where trolling is conceptualised as a disruptive online behaviour intended to provoke and distress others for one’s own amusement [11–13]. Early conceptualisations of trolling outlined four main characteristics of the online behaviour: Deception, aggression, disruption, and success [14]. The disruptive characteristic of trolling distinguishes this behaviour from other online antisocial behaviour, such as cyberbullying [9, 14]. Trolls deliberately deceive others by pretending to be a sincere member of an online community, when their real intention is to trigger conflict and cause disruption for the purposes of their own amusement [14]. If their aggressive or malicious behaviour goads others into retaliating, then their trolling is considered a success [14].

Online behaviours that characterise trolling are increasingly common; for example, The Australia Institute reported that 39% of the adult population have experienced online harassment [15]. It is also likely that some trolling behaviours (e.g., undirected swearing) are more prevalent than others (e.g., targeted harassment) [16]; however, given that the very nature of trolling is deceptive [14], it is likely that estimates of trolling behaviours are conservative. The occurrence of trolling is concerning, as the behaviour can have negative impact for both the troll and their target. In instances where trolling behaviour is considered menacing, harassing, or offensive, the troll may experience legal ramifications. Such ramifications range in severity from being directed to remove the material, civil penalties, and in extreme cases possible imprisonment, with recent legislation (e.g., *Australian Online Safety Act 2021*) strengthening existing online safety laws. Targets of trolling report experiencing negative psychological consequences such as distress and lower self-esteem [17], and increases in suicidal ideation and self-harm behaviours [18]. The potentially high prevalence and negative impact of trolling highlight the importance of ongoing research exploring this antisocial online behaviour. Further, we posit research attention should be directed towards populations that may be at increased risk of both being trolled and trolling others—in particular, adolescents [8].

### Adolescents and trolling

Adolescence is a significant biological, social, and psychological developmental period [19]. During this developmental period, adolescents adjust to new challenges and develop new skills, responsibilities, and intimate relationships [19]. Although adolescence is a time for positive growth, it is also a period of new challenges and risks [20]. The neurobiological changes that occur during this developmental period [21] influence the adolescent to seek novelty, arousal, and excitement [22] and to take more risks [23]. It is this novelty-seeking and risk-taking that renders adolescence, although a time of exploration and growth, also a time of vulnerability [21, 22].

Social media provide social, educational, and mental health benefits to adolescents [1–3]; however, social media can also present threats to adolescents’ mental health and wellbeing through experience of antisocial online behaviour [24]. Given the increased frequency of time adolescents spend on social media [25], ongoing research exploring adolescents’ online experience and behaviour is crucial. Importantly, adolescents may be especially vulnerable to experiencing adverse online experiences as they no longer receive the ongoing parental supervision they did as children, yet have not entirely developed an understanding of behaviour and consequences [26]. As trolling has been associated with increased dysfunctional impulsivity [27], and adolescence is also a period of elevated impulsive behaviour [28], it follows that adolescents may be particularly likely to engage in trolling behaviours.

### Personality and trolling: Psychopathy and sadism

Previous multi-disciplinary research exploring predominantly adult populations has linked certain personality traits with trolling [11, 29, 30]. Reliable, positive correlations have been found between the Dark Tetrad traits of personality and trolling behaviours. The Dark Tetrad [31] comprises the four related, but distinct, nonclinical personality traits of Machiavellianism, sadism, narcissism, and psychopathy, traits that are characterised by emotional and social coldness, duplicity, self-promotion, and aggressiveness [27]. In adult samples, although narcissism and Machiavellianism share positive correlations with trolling behaviours [11, 27, 29], only psychopathy and sadism have consistently emerged as strong, positive predictors of trolling [9, 11, 29]. To date, it is unknown if the utility of psychopathy and sadism to predict trolling behaviours can be replicated in adolescent samples. It should be noted that we recognise the preference to refer to the construct of psychopathy as ‘psychopathic traits’ in the absence of formal assessment; however, as the current study sits within the wider Dark Tetrad of personality [31] literature, which adopts the term psychopathy, we opt to remain consistent with this literature.

Psychopathy is characterised by diminished empathy, impulsivity, thrill-seeking [32], interpersonal manipulation, grandiosity, and emotional shallowness [33]. As a personality trait, psychopathy shows stability from early adolescence into adulthood, though previous research has shown a tendency for psychopathy levels to be lower in pre-teen years, rising through secondary schooling and lower again at the end of adolescence (age 17) [34]. Psychopathy in adolescents has been shown to be related to problems with emotional processing, deficits in behavioural inhibition, and increased impulsivity [34–36]. Further, adolescents with high psychopathy engage in more online aggression [37]. Given the positive associations that exist between trolling and disinhibition [38] and trolling and dysfunctional impulsivity [27], there is rationale to expect that adolescents with high psychopathy will engage in increased incidence of trolling.

Nonclinical sadism is characterised by the enjoyment of causing others harm, and those with high sadism manipulate others using fear and intimidation [11]. Individuals with high levels of sadism are considered to derive pleasure from hurting others [39], and sadism is a strong, positive predictor of trolling in adult samples [29, 40, 41] and aggressive online behaviour in an adolescent sample [42]. Sadistic traits have been demonstrated to be strongly related to antisocial behaviours in adolescents and are a useful factor in understanding socially aversive behaviours in young people [31]. In the current study, we sought to establish if psychopathy and sadism could predict trolling behaviours in an adolescent sample.

### Other factors relevant to adolescence

Several other factors have also been implicated as potentially playing a role in trolling behaviours. In adult samples, low self-esteem has been related to increased trolling incidence [7, 13]. The links between self-esteem and trolling have not yet been explored in adolescent populations and therefore whether similar effects would be expected for younger populations is unknown. Due to the cognitive and social changes occurring during adolescence, it is generally a period in which awareness and concern with self-image become important to life decisions [43]. Self-esteem has been shown to be generally lower in adolescence and demonstrated to be a key vulnerability factor for negative outcomes such as depression and social problems [44]. Further, self-esteem can affect how a young person engages in identity formation [45] and develops social relationships [46], key developmental challenges of adolescence that could also potentially influence trolling behaviours. Therefore, there is rationale to expect that in an adolescent sample, the role of self-esteem in trolling may present differently to an adult sample.

In addition to self-esteem, empathy has been associated with perpetration of trolling behaviours in a sample of adults [9] and may also relate to incidence of trolling in an adolescent sample. Empathy is a multidimensional construct [47], comprising affective (the ability to internalise, share, and experience others’ emotions), and cognitive (the ability to recognise and understand others’ emotions) dimensions [48]. For adults, low levels of affective empathy have been correlated with increased trolling; however, cognitive empathy shares no relationship [9]. This indicates that adults who troll are less likely to share the emotional experience of the pain they cause their targets, though the more analytic form of empathy is unrelated to the online behaviour. While these findings contribute to the psychological profile of adults who troll, research is yet to determine whether this profile holds true for adolescents.

To theorise the role that empathy may play in adolescents’ who troll, we draw on the extant literature exploring adolescents’ empathy and other antisocial behaviour. In children and adolescent samples, low cognitive and affective empathy have been linked to increased incidence of bullying, with the magnitude of effect stronger for affective empathy [49]. Comparatively, in a sample of preadolescents, only low cognitive empathy—not low affective empathy—was linked to increased bullying incidence [50]. The authors speculated that preadolescents with low cognitive empathy may not have a good understanding of others’ emotions, which impacts their own ability to self-regulate aggressive behaviour [50]. The associations between empathy and bullying behaviours may also demonstrate differential patterns across gender. In a sample of adolescents, low perspective taking (i.e., cognitive empathy) and low empathic concern (i.e., affective empathy) were associated with increased bullying for boys, but *high* perspective taking was associated with increased bullying for girls, with no relationship for empathy concern [51]. A lack of empathy in adolescents (termed callous-unemotional traits), has been linked to adult psychopathy [34], and adolescents with high levels of callous-unemotional traits are more likely to bully others [52].

Although limited research has explored the role of cognitive empathy in antisocial online behaviour, we can extrapolate from studies exploring emotional intelligence to understand the role of cognitive empathy. Like cognitive empathy, emotional intelligence is defined as the ability to perceive, recognise, understand, and express others’ emotions [50]. Emotional intelligence has also been linked to bullying, with varying relationships depending on how emotional intelligence is conceptualised [50]. When conceptualised as cognitive ability, high social intelligence is associated with more sophisticated forms of bullying, and low social intelligence is associated with more direct and physical forms of bulling [53]. When conceptualised as a personality trait, low emotional intelligence is associated with increased frequency of bullying [50]. Thus, while much of the research exists in adult trolling research, or youth bullying contexts more generically (i.e., not specific to trolling), it is clear that the role of cognitive and affective empathy in antisocial behaviours during adolescence may not be straightforward. This may be further compounded by the fact that empathy is still developing during adolescence, with adolescents showing lower cognitive empathy and affective empathy compared to adults [54]. The potential role of empathy in adolescent trolling appears important, though due to developmental considerations, may not be the same as that in adults.

A final theoretical and developmental perspective of importance is adolescents’ attunement to social interactions, that is theorised to increase their sensitivity to social rewards [55]. Callous unemotional traits, such as lack of empathy, have been shown to be negatively aligned with prosocial interactions and positively related to enjoyment of antisocial rewards [56]. Social rewards are described as the social stimuli an individual experiences as rewarding [57]. Those who experience reward via antisocial interpersonal interactions (also termed negative social potency [57]) are likely to enjoy exerting negative social influence [29], and adults with high negative social potency have been found to engage in more trolling [29, 58]. For adolescents, high reward-seeking behaviour has been linked to increased antisocial behaviour [59, 60]. Based on findings in adult samples, and the tendency for reward systems to be linked to antisocial behaviour in adolescence, it is likely adolescents with high negative social potency may engage in more trolling. In summary, this review of the literature demonstrates that personality, psychological traits, and social rewards, important to trolling in adults are likely to also play a role in adolescents’ trolling; however, to date, no research has examined this. Psychopathy, sadism, self-esteem, empathy (cognitive and affective), and negative social potency have all correlated with increased trolling incidence in adult samples and have been linked with other forms of antisocial behaviour (e.g., bullying and cyberbullying) within an adolescent population.

### Aims and hypotheses

In the current study, we aimed to explore adolescents’ experience of trolling, including how often they are trolled and how often they troll others. Further, we aimed to explore the psychological profile of adolescents who troll, specifically, by examining the utility of personality traits (i.e., psychopathy and sadism), self-esteem, empathy (cognitive and affective), and social rewards (specifically negative social potency) to predict trolling in a sample of Australian adolescents. Given the lack of research on trolling and adolescents, this nature of this study is somewhat exploratory. Still, based on previous results in adult samples, we hypothesise that for adolescents, high psychopathy, high sadism, low self-esteem, low cognitive empathy, low affective empathy, and high negative social potency will predict more trolling. By identifying the potential characteristics and profile of adolescents who troll, we aim to inform the development of intervention and prevention strategies to reduce the impact of antisocial online behaviours.

## Methods

### Participants

A total of 157 Australian adolescents completed the study. Participants were aged 13–18 years old with an average age of 15.58 years (*SD* = 1.71). Of participants, 64 (40.8%) identified as male, 91 (58%) identified as female, and 1 (0.6%) identified as non-binary (1 participant did not respond).

### Measures

Participants completed an anonymous online questionnaire comprised of demographics and a series of measures in the order presented below. All measures excluding assessment of trolling behaviours have been validated for use in adolescent samples.

#### Demographics

Participants were asked to provide their age, postcode or city/town, and gender. To understand participants social media usage, questions were asked to determine how much time they spend on social media, which social media sites they currently use, how they access their social media accounts, what information they include on their social media profiles, and why they use social media. An overview of participants social media use can be found in S1 Table.

#### Global Assessment of Internet Trolling-Revised (GAIT-R [9])

The GAIT-R (α = .73) was included in the current study to assess participants’ trolling behaviours. Minor changes in the wording of two items were made to better align with an adolescent sample. The GAIT-R is comprised of 8 self-report items (e.g., “Some of my funny Internet posts/comments could be considered offensive by others”) and participants respond to items on a 5-point Likert scale (1 = Strongly Disagree, 5 = Strongly Agree). One item is reverse scored. After recoding, responses are summed for total scores with higher scores indicating more trolling behaviours.

#### Experiences of trolling

Participants were provided with an operational definition of Trolling and asked to respond on a dichotomous “yes/no” scale to “During the past year, did anyone ever troll you on the Internet?”. The operational definition read:

Trolling is internet slang for a person who intentionally starts arguments or upsets others by posting comments that will get a reaction. These people are referred to as “trolls”. Trolls like to provoke and upset other people online, with the intention to cause some harm. A troll might leave rude comments in a public comment section, with the purpose of grabbing the attention of other visitors and disrupting the discussion that would otherwise be about the page’s content. The comments might include content that is hateful, racist, sexist, or disrespectful.

A “yes” response further prompted participants “How many different times did someone troll you on the Internet in the last year (for example, at different times, by different people, or for different reasons)?” with response options ranging from “1” to “6 or more”. Participants then responded on the same scale as outlined above about their experience of engaging in the behaviour (i.e., “…did you ever troll someone on the Internet… how many different times did you troll others on the Internet…”). The format of these items was based on the Bullying and Cyberbullying Scale for Adolescents [61].

#### Adolescent Measure of Empathy and Sympathy (AMES [47])

The AMES was included to measure adolescents cognitive and affective empathy. The AMES also includes a subscale of sympathy, however, this was not included in the current study. The cognitive empathy subscale (α = .83) includes four items (e.g., “I can often understand how people are feeling even before they tell me”) and the affective empathy subscale (α = .80) includes four items (e.g., “When my friend is sad, I become sad too”). Participants respond on a 5-point Likert scale (1 = Never, 5 = Always). Responses are summed for total scores with higher scores indicating higher levels of empathy.

#### Rosenberg Self-Esteem Scale (RSES [43])

The RSES (α = .86) was used to measure adolescent global self-esteem. Participants respond to 10 self-report items (e.g., “I take a positive attitude toward myself”) on a 4-point Likert scale (1 = Strongly Disagree, 4 = Strongly Agree). Five items are reverse scored and after recoding, responses are summed for total scores with higher scores indicating higher levels of overall self-esteem.

#### Youth Psychopathy Traits Inventory—Short Version (YPI-S [62])

Adolescents’ psychopathy was assessed with the YPI-S (α = .86), an 18-item self-report measure. The YPI-S includes three subscales (dimensions) of psychopathy: Interpersonal (e.g., “It’s easy for me to manipulate people”), affective (e.g., “I think that crying is a sign of weakness, even if no one sees you”), and behavioural (e.g., “I consider myself as a pretty impulsive person”). Respondents indicate how strongly statements apply to them on a 4-point Likert scale (1 = Does not apply at all, 4 = Applies very well). Responses to subscales are summed for a total score.

#### Social Rewards Questionnaire (SRQ [57]-)

The negative social potency subscale (α = .84) of the SRQ was included to measure the degree adolescents experienced reward when engaging in antisocial interpersonal interactions (i.e., negative social potency). The subscale includes 5 items (e.g., “I enjoy making someone angry” and “I enjoy tricking someone out of something”) and participants respond to items on a 6-point Likert scale (1 = Strongly Disagree, 6 = Strongly Agree). Responses are summed for total scores, with higher scores indicating higher negative social potency.

#### Short Sadistic Impulse Scale (SSIS [63])

The SSIS (α = .88) was used to measure adolescents’ level of everyday sadism. The SSIS is comprised of 10 self-report items (e.g., “I have hurt people because I could”) and participants indicate their agreement to items on a 5-point Likert scale (1 = Strongly Disagree, 5 = Strongly Agree). One item is reverse scored. After recoding, responses are summed for total scores with higher scores indicating higher levels of sadism.

### Recruitment

Ethical approval was granted by the University of Southern Queensland Human Research Ethics Committee with Approval No. H17REA260. Participants were recruited by advertising through social media accounts (both personal accounts of the researchers and their universities’ accounts), first year undergraduate psychology courses at the authors’ institutions, and word of mouth. Phase 1 of recruitment also included contacting the principals of High Schools in the greater Brisbane and Toowoomba areas, requesting they share the advertisement in their School newsletters (or similar).

Inclusion criteria for participation was age (between 13–18 years) and that social media was used in a typical week. The link in the study advertisement directed participants to details of the study. Written consent was obtained from all adolescent participants by clicking the *Next* button at the bottom of the page containing detailed information about the anonymous study. The Children’s Online Privacy Protection Act recommends children under the age of 13-years do not use social media applications. As the target cohort was older than this, and able to use social media, in addition to the low-risk nature of the study, parental permission was not obtained for participants before joining the study. Although not anticipated, to minimise any risks of participation in the project, information and contact details were included at the bottom on each survey page in case the issues raised in the questionnaire created uncomfortable or distressing feelings. Participants could also choose to withdraw at any time during the study prior to the final submission page by not submitting the survey or closing their browser. After consent was provided, the study became accessible. Tips promoting online safety were included on the final page of the survey. Completion of the survey in its entirety took approximately 30 minutes.

There were three phases of recruitment. Each phase included the same recruitment process excluding incentives offered for participation, and the contacting of School principals (isolated to Phase 1). Phase 1 took place between 1^st^ June, 2021 and 26^th^ July, 2021. During this phase, participants were given the option of entering a draw to win one of 10 Ultimate Student gift cards valued at $50 (sent electronically to the winners of the draw) or receive course credit (if enrolled in an eligible university course). Once participants completed the study, they were directed to a second, unlinked survey (allowing data to remain deidentified) to enter their email (Phase 1) or postal (Phases 2 and 3) address, for the draw, or provide the relevant details for course credit. Phase 1 resulted in the recruitment of 68 participants.

To achieve an adequate sample size, two additional recruitment phases were added. Phase 2 took place between 2^nd^ August, 2021 and 8^th^ September, 2021 and resulted in the recruitment of 46 participants. Phase 3 took place between 13^th^ June, 2022 and 15^th^ August, 2022 and resulted in the recruitment of 45 participants. Phase 3 was unplanned but necessary to increase the sample size after detailed data screening of Phases 1 and 2 indicated the likely presence of bot generated data (explored in detail in S1 Appendix). Participants in Phases 2 and 3 were offered a $15 Woolworths Essential gift card (posted to participants) for completion of the survey.

Responses for Phases 1 and 2 were collected through UniSQ Survey Tool and data for Phase 3 was collected through Qualtrics. A CAPTCHA (i.e., Completely Automated Public Turing Test to tell Computers and Humans Apart) was included in Phase 3.

### Design and statistical analysis

The study was a correlational, cross-sectional design with the predictor variables of psychopathy, sadism, self-esteem, cognitive empathy, affective empathy, and negative social potency. The criterion was trolling (as measured by the GAIT). Following guidelines of previous studies [29, 58], gender was included as a control variable. The hypothesis was tested with a 2-Step Hierarchical Multiple Regression Analysis with gender entered at Step 1 and the predictor variables entered at Step 2. Analyses were run using SPSS Version 25. For 7 variables, an a priori power analysis using G Power [64] (Liner multiple regression: Fixed model, R^2^ deviation from zero) with power at .95, an effect size of .15, and alpha at .05, indicated a sample size of 153 was required for adequate power and this was satisfied.

## Results

### Descriptive statistics

Based on the operational definition of trolling provided, 24.2% of participants indicated they had been trolled in the previous year (by gender 28.1% boys and 19.8% of girls), and 13.4% of participants indicated they had trolled others in the previous year (by gender 28.1% of boys and 3.3% of girls). A frequency table for adolescents who had been trolled and who had trolled others can be seen in Table 1.

**Table 1:** Frequency table for trolling.

	Had been trolled in the past year		Had trolled others in the past year	
	*n*	%	*n*	%
1 time	6	3.8	5	3.2
2 times	11	7	5	3.2
3 times	15	9.5	8	5.1
4 times	1	.6	1	.6
5 times	-	-	-	-
6 or more times	5	3.2	2	1.3

Supplementary analyses using the descriptive trolling data is presented in S2 Appendix. Detailed data screening and assumption testing for the primary analyses are presented in S1 Appendix.

Descriptives (total and by gender) can be seen in Table 2, and bivariate correlations are presented in Table 3. Bivariate correlations for boys and girls separately are presented in S2 Table. For the bivariate correlations, we included age to determine whether it should be included as a control variable in the regression analysis.

**Table 2:** Descriptive statistics for predictor variables of psychopathy, sadism, self-esteem, cognitive empathy, affective empathy, and negative social potency and criterion of trolling.

	Total		Boys		Girls				
	*M* (*SD*)	Min—Max	*M* (*SD*)	Min—Max	*M* (*SD*)	Min—Max	D	*t*	*d*
Psychopathy	39.12 (8.51)	20–62	40.14 (8.89)	20–62	38.38 (8.28)	21–59	1.76	1.26	.21
Sadism	18.24 (7.07)	10–40	20.13 (8.30)	10–40	16.85 (5.80)	10–33	3.28	2.73**	.47
Self-esteem	23.62 (5.61)	10–39	23.39 (4.96)	10–37	23.70 (6.02)	10–39	-0.31	0.95	-.06
Cognitive empathy	14.87 (2.64)	7–20	14.00 (2.66)	7–20	15.53 (2.47)	9–20	-1.52	-3.67***	-.60
Affective empathy	12.78 (3.11)	4–20	12.30 (2.65)	4–17	13.11 (3.39)	4–20	-0.81	-1.67	-.26
Negative social potency	11.28 (5.54)	5–30	13.03 (6.35)	5–30	10.11 (4.59)	5–24	2.92	3.15***	.54
Trolling	16.25 (5.52)	8–36	18.06 (6.79)	8–36	14.98 (4.06)	8–29	3.08	3.53***	.58

Note.

*p < .05,

**p < .01,

***p < .001;

boys *n* = 64, girls *n* = 91; D = mean difference; *d* = Cohen’s *d*

**Table 3:** Bivariate correlations for age, gender, psychopathy, sadism, self-esteem, cognitive empathy, affective empathy, and negative social potency, and trolling.

	1.	2.	3.	4.	5.	6.	7.	8.
1. Age	-							
2. Gender	-.03	-						
3. Psychopathy	-.06	-.10	-					
4. Sadism	-.04	-.23**	.52***	-				
5. Self-esteem	-.06	.03	.08	.08	-			
6. Cognitive empathy	-.06	.29***	-.13	-.11	.02	-		
7. Affective empathy	.02	.13	-.19*	-.16*	-.02	.29***	-	
8. Negative social potency	.05	-.26***	.57***	.70***	.08	-.21**	-.15	-
9. Trolling	.11	-.27*	.43***	.39***	.06	-.18*	-.04	.53***

Note.

*p < .05,

**p < .01,

***p < .001;

gender coded as 0 = boy, 1 = girl; multicollinearity not detected

As can be seen in Table 2, there were significant positive correlations between trolling and psychopathy, sadism, and negative social potency. There were significant negative correlations between trolling and gender (male) and trolling and cognitive empathy. Affective empathy did not significantly correlate with trolling, but due to shared variance between cognitive empathy and affective empathy, both variables were included in the regression analysis. Due to a lack of a bivariate correlation, age was not included as a control variable in the regression analysis.

### Hierarchical multiple regression analysis

A 2-Step Hierarchical Multiple Regression Analysis was run to predict trolling with gender entered at Step 1 and psychopathy, sadism, self-esteem, cognitive empathy, affective empathy, and negative social potency entered at Step 2. At step 1, gender explained a significant 6.9% (R^2^ adjusted) of variance in trolling, R^2^ = .08, *F*(1, 153) = 12.45, *p* < .001. At step 2, psychopathy, sadism, self-esteem, cognitive empathy, affective empathy, and negative social potency explained an additional significant 26.3% (R^2^ change) of variance in trolling, and this change was significant, *F*change(6, 147) = 9.74, *p* < .001. As a total model, the variables explained a significant 30.7% (R^2^ adjusted) of variance in trolling, R^2^ = .34, *F*(7, 147) = 10.73, *p* < .001, with a large effect size of *ƒ*^2^ = .52. Coefficients are presented in Table 4.

**Table 4:** Coefficients for gender, psychopathy, sadism, self-esteem, cognitive empathy, affective empathy, and negative social potency regressed on criterion of trolling.

	B [95% LL, UL]	*SE*	*β*	*t*	*p*
*Step 1*					
Constant	18.06 [16.74, 19.39]	0.67			
Gender	-3.08 [-4.81, -1.36]	0.87	-.27	-3.53	.001
*Step 2*					
Constant	6.66 [-0.72, 13.51]	3.47			
Gender	-1.77 [-3.37, -0.16]	0.81	-.16	-2.17	.031
Psychopathy	0.14 [0.03, 0.25]	0.06	.22	2.55	.012
Sadism	-0.02 [-0.18, 0.13]	0.08	-.03	-0.31	.756
Self-Esteem	0.03 [-0.10, 0.17]	0.07	.03	0.43	.668
Cognitive Empathy	-0.10 [-0.40, -0.21]	0.16	-.05	-0.63	.530
Affective Empathy	0.15 [-0.10, 0.40]	0.13	.09	1.21	.220
Negative Social Potency	0.39 [0.19, 0.59]	0.10	.39	3.76	.001

*Note*. Gender coded as 0 = boy, 1 = girl

As can be seen in Table 4, in the final model gender was a significant, negative predictor, with boys trolling more than girls, and psychopathy and negative social potency were significant, positive predictors. We were interested why, given the strong, positive bivariate correlation, sadism did not emerge as a significant predictor of trolling. We speculated that perhaps the variance shared between sadism and psychopathy (*r*^2^ = 27%) and sadism and negative social potency (*r*^2^ = 49%) was captured in the regression model, rendering sadism a nonsignificant predictor of trolling. To test this speculation, we ran two partial correlations between sadism and trolling controlling for psychopathy and negative social potency, respectively. When controlling for psychopathy, sadism and trolling still shared a significant, positive correlation, *r*(154) = .21, *p* = .009, however, when controlling for negative social potency, sadism and trolling were no longer correlated, *r*(154) = .02, *p* = .762.

## Discussion

In the current study, we explored adolescents’ experience of trolling, including how often they are trolled and how often they troll others. Further, we aimed to replicate adult research that has constructed a psychological profile of the Internet troll by exploring the utility of personality traits (psychopathy and sadism), self-esteem, empathy (cognitive and affective), and social rewards (specifically negative social potency) to predict perpetration of trolling in a sample of Australian adolescents. We predicted that high psychopathy, high sadism, low self-esteem, low cognitive empathy, low affective empathy, and high negative social potency would predict more trolling, and results supported this hypothesis.

### Adolescents and trolling: A descriptive analysis

We documented the frequency that Australian adolescents reported both being trolled and having trolled others. This documentation is a particularly novel contribution of the current study, as we provided adolescents with an operational definition of trolling, which has not previously occurred. Given the discrepancies that exist regarding defining trolling amongst both researchers and the general population [10, 65], by providing an operational definition, we attempted to explicitly capture the occurrence of this behaviour. Here, the operational definition captures the intention to trigger conflict and cause disruption—elements that may not be adequately captured by the broad measurement of the GAIT-R. In our study, approximately one quarter of Australian adolescents (24.2%) reported they had been targeted by trolling in the previous year, and 13.4% reported they had trolled others in the previous year. More boys than girls reported being trolled (28.1% of boys and 19.8% of girls), and trolling others (28.1% of boys and 3.3% of girls). We also explored the number of times Australian adolescents had been trolled and trolled others in the previous year, with most participants experiencing or perpetrating trolling three (13.6% and 8.1%, respectively) or two times (10% and 5.1%, respectively). These occurrences of trolling experienced by Australian adolescents are somewhat reflective of online behaviours that characterise trolling reported in adult populations, albeit less, with over one in three Australian adults being the target of online harassment [15]. Unfortunately, rates of trolling others are unclear in Australian adults and direct comparisons are not able to be drawn.

The rates of trolling experienced by Australian adolescents in this sample are similar, albeit slightly higher, than the experience of cyberbullying, as recent reports show approximately 20% of Australian adolescents experience cyberbullying [15]. Although the prevalence statistics of cyberbullying others for Australian adolescents is unclear, one study in Florida found 12% of adolescents admitted to cyberbullying others [66], a similar proportion to trolling reported in the current study. The increased likelihood of adolescents to experience trolling, compared to cyberbullying, highlights the importance of research exploring trolling in this particularly vulnerable group.

We found that the most popular social media platforms used by participants were Instagram (77.1%), YouTube (71.3%), and Snapchat (65.6%), a finding consistent with recent research [67]. While we did not explore trolling experiences on individual social media platforms, we did examine potential differences in the time spent on social media per day and the total number of social media platforms used, between those who had trolled others and those who had not trolled others, and between individuals who had been trolled and individuals who had not been trolled. All comparisons were non-significant, indicating that irrespective of trolling experiences, there were no differences in the duration of time spent on social media per day or the number of social media platforms used. We did, however, find a moderately positive correlation between being trolled and trolling others, that is, our findings suggest that people who have been trolled are more likely to troll others.

### Adolescents and trolling: Building a psychological profile

In adult samples, psychopathy, sadism, self-esteem, empathy, and negative social potency have all demonstrated relationships with trolling. In this study, these constructs, plus gender, cognitive empathy, and affective empathy, explained 30.7% of variance in adolescents’ trolling. When examining the specific predictors, there was some replicability of previous findings regarding predictors of trolling in adult sample; however, also some notable differences.

We found that for adolescents, high psychopathy both correlated with and uniquely predicted increased incidence of trolling. This finding is consistent with results of adult samples [9, 13, 30], and in line with previous research that found adolescents with high psychopathy were more aggressive online [37]. For adolescents, the combination of a grandiose sense of self-worth and manipulative behaviour (i.e., interpersonal psychopathy), poor behavioural control and delinquency (i.e., behavioural psychopathy), and a lack of remorse and guilt (i.e., affective psychopathy) predicts higher trolling.

Also similar to adult samples [29, 58], high levels of negative social potency both correlated with and emerged as a unique predictor of adolescents’ trolling. Individuals with high negative social potency experience feelings of enjoyment as a result of antisocial interpersonal interactions [56]. As previous research has linked high negative social potency to increased trolling in adult samples [29, 58], and reward-seeking to antisocial behaviour in adolescence, it follows that this construct would emerge as a strong, positive predictor of trolling behaviours for adolescents.

Adolescents’ sadism was a significant, positive correlate of trolling behaviours—a finding also consistent with adult samples [29, 40, 41, 58]. At a bivariate level, it appears that adolescents who enjoy causing others physical and/or psychological harm are more likely to troll. This finding is also consistent with research on sadism and cyber aggression with adolescents [42], however, in the current study, sadism did not emerge as a unique predictor of trolling in the shared variance model—a finding that varies from adult samples [e.g., 58] and research with adolescents’ aggressive online behaviour [42]. As the current study is the first to explore trolling incidence in an adolescent sample, our interpretation of this finding is somewhat speculative. Still, we speculated that perhaps the overlapping variance of sadism, psychopathy, and negative social potency could account for the lack of this unique, predictive utility. When controlling for psychopathy, sadism and trolling remained significantly, positively correlated. When controlling for negative social potency, however, sadism and trolling no longer correlated. We posit that this result highlights a crucial difference between adolescent and adult samples regarding trolling. For adults, both sadism and negative social potency are unique predictors of trolling—indicating that adults who enjoy causing others harm and who are motivated by antisocial rewards (negative social potency) are more inclined to troll [29]. For the adolescent, however, although there may be elements of the enjoyment of harming others (i.e., sadism), this variance is rendered nonsignificant when the enjoyment of antisocial rewards is captured. Put simply—adolescents troll because they enjoy engaging in antisocial interpersonal interactions, adults also troll for this reason, and to cause harm.

In the current study, adolescents’ cognitive empathy was a significant, negative correlate of trolling behaviours. Affective empathy was not correlated with trolling, and neither dimension of empathy emerged as a unique predictor of adolescents’ trolling in the shared variance model. This pattern of results for empathy and trolling appears qualitatively different to adults who troll, as past research has shown adults’ cognitive empathy has no significant bivariate relationship with trolling, and adults with low levels of affective empathy more likely to troll [9]. As the current study is the first to explore these dimensions of empathy in relation to adolescents’ trolling, interpretation of these results is speculative. Still, it appears that the lack of ability to recognise and understand others’ emotions (i.e., cognitive empathy) in adolescence, rather than the ability to internalise and experience others’ emotions (i.e., affective empathy), may play a role in trolling behaviour. These findings align with previous suggestions that adolescents with high levels of cognitive empathy may have a better understanding of others’ emotions and the impact of bullying, and as a result bully less [51]. The bivariate findings also somewhat align with the association between a lack of empathy in adolescents and increased incidence of bullying others [52]. The current findings indicate that unlike adult samples, where low affective empathy plays an important role in trolling behaviour, low cognitive empathy may be important in understanding adolescent trolling behaviour. We recommend that future researchers exploring trolling across adolescents and adults further explore why these forms of empathy demonstrate different patterns for these age cohorts. Importantly, this finding indicates that interventions to manage online antisocial behaviour, such as empathy training [68], should be differentiated for adult and adolescent cohorts.

Unlike adult samples where self-esteem shares a negative bivariate relationship with trolling [13], self-esteem did not emerge as a significant correlate, or predictor, of trolling behaviour in adolescents. Self-esteem has been shown to increase in adolescence, a critical developmental stage [69]. Although self-esteem can affect how a young person engages in identity formation [45] and develops social relationships [46], it did not predict trolling behaviours. As low self-esteem has previously been linked to adolescents cyberbullying others [70, 71], results of the current study further distinguish trolling from cyberbullying, highlighting the need for continued research in adolescent and adult samples to explore trolling as an online antisocial behaviour separate to cyberbullying. This distinction may be particularly important for prevention and education interventions, which will likely target different factors for adolescent trolling and cyberbullying.

Although the variables of gender and age were not specific variables of interest, they warrant consideration. Similar to adult cohorts [9, 13], age was not a significant correlate of trolling for adolescents. We found that gender was a significant predictor of trolling behaviours, with boys more likely than girls to engage in trolling. This finding is in line with past research in adult samples which has found men are more likely to perpetuate trolling behaviours compared to women [9, 29, 40], and previous findings that, compared to adolescent girls, adolescent boys are more likely to cyberbully others [72].

Of interest, a recent study found that compared to adolescent girls, adolescent boys who experienced cyberbullying increased their likelihood of cyberbullying others [73]. A trolling cycle, whereby the victim becomes a subsequent troll, sometimes in the spirit of revenge, has also been documented in adult samples [74]. Although outside of the scope of the current study, we recommend future researchers exploring both adolescents’ and adults’ trolling consider the victimization-perpetration nexus and explore the potential moderating role of gender. This seems particularly pertinent combined with our finding regarding the significant, positive correlation between adolescents trolling and being trolled (in addition to the supplementary analyses split by gender presented in S2 Table). Such research could highlight an important area for intervention, particularly for adolescent boys and girls; specifically, risk management for those targets as they may be a group likely to engage in this antisocial online behaviour.

### Implications

The findings of this study indicate ongoing research, education, and skills training is necessary to better understand adolescents’ trolling. Given that the experience of trolling is associated with significant negative psychosocial outcomes for both the troll (e.g., legal ramifications) and their target (e.g., distress [17] and self-harm behaviours [18]), there is impetus to better understand factors contributing to the onset of the behaviour, and to identify and develop appropriate methods for reducing the occurrence and impact of trolling on adolescents.

Mindful that we did not examine an education or intervention program, in line with previous research exploring individual differences and trolling [29], these findings may have implications for programs designed to prevent or manage trolling in adolescence. For example, interventions could include information on the psychology of trolls. Specifically, these programs could educate on the motivations behind trolling behaviours, empowering adolescents to recognise trolling behaviours and respond in a manner that diffuses the situation, such as not engaging and reinforcing the troll. Additionally, from the perspective of the potential adolescent who may troll, interventions could focus on skills training for developing empathy and responsibility for their actions, as well as education and training in improving ability to recognise and understand others’ emotions, particularly understanding how trolling behaviours can hurt others.

### Limitations and future directions

A potential limitation of the current research is the sampling procedure. More specifically, participation was voluntary and anonymous and therefore susceptible to selection bias. For example, adolescents who had previously been trolled or who had trolled others may have been more likely to complete the survey. Data was also collected at three separate time points spanning a timeframe of approximately 14.5 months. As part of the data screening (outlined in S1 Appendix), analyses were performed to compare participant scores across each of the recruitment phases on the predictor and criterion variables. Analyses indicted a significant difference, with self-esteem scores higher in Phase 3 relative to the other phases. Given self-esteem was not implicated in any significant bivariate or predictive relationships, this was not considered problematic.

Another limitation of the current study was the absence of strategy to prevent and detect bot activity in Phases 1 and 2 of data collection (for a detailed overview of bot screening see S1 Appendix). In response to detecting bot activity and based on recommendations from recent research [75], we implemented a number of bot prevention and detection strategies in Phase 3, such as the inclusion of a CAPTHCA and proactively monitoring time stamps of survey onset and completion. Interestingly, and concerningly, the bot generated data did pass other recommended data screening assessments (e.g., Mahalanobis distance) [76]. Such a finding has important implications for future research adopting this as a single strategy for identifying bot activity. Based on our assessment, a combination of time of survey commencement, completion, and location information that was the best indication of bot generated data. Based on our experience, and screening indicators, we strongly recommend that future researchers adopt a combination of bot prevention and detection strategies to ensure integrity of data and research.

The context in which the survey has taken place also needs to be considered. During all phases of data collection, the world was experiencing a global pandemic that impacted Australian adolescents’ lives and social media usage. At times there was reduced opportunities for in-person interaction with peers with the implementation of social distancing guidelines, lockdowns, and online learning. In adult samples, perpetration of antisocial online behaviour, specifically cyberbullying, increased during the pandemic [77]. As such, it is possible that adolescents’ perpetration of antisocial online behaviour also increased during the pandemic, potentially skewing the current findings. Still, it is worthwhile to note that some adolescent samples reported no change in experiencing cyberbullying during the pandemic [78]. Importantly, the current findings largely reflect trends of previous research conducted pre-pandemic. As such, we assert that although the context of the pandemic is important when interpreting the results, the findings are robust and extend beyond the context.

To better understand trolling behaviours of adolescents and develop targeted interventions, future research should further examine the specific platforms and contexts this behaviour occurs in. It would be of interest to determine on which social media platforms adolescents experience the greatest incidence of trolling, and if such platforms potentially moderate the relationship between gender, psychological constructs (e.g., personality and self-esteem) and trolling. Qualitative research with adolescents regarding the experience of trolling would also be of value. More specifically, in-depth examinations of how trolling occurs, including victim-perpetrator responses, would greatly assist in understanding the antecedents that trigger the behaviour and the consequences that contribute to maintaining Internet trolling behaviours. Additionally, a longitudinal focus on studying adolescents over time would provide insight into the long-term effects of trolling, and for those adolescents who are also trolls, understanding on the evolution of a troll from adolescence to adulthood. This will also provide insight into additional areas for intervention, by highlighting where education strategies should be targeted and what skills are required of both the target and the “troll” to manage or halt the interaction.

Given the complexity of trolling, we also recommend that future research consider exploring the intentions associated with trolling behaviour. For example, does the troll intend to entertain (i.e., kudos trolling [79]) or abuse and harm others? It is likely that differential patterns will emerge for personality traits and these intentions—for example, sadism may be better predictive of the abusive form compared to the entertaining form. Future research exploring these intentions could elucidate nuances associated with different trolling behaviours and the nomological network of associated psychological characteristics. Further, although outside the scope of the current study, future research might also consider exploring potential mediation and moderators of trolling for both adolescents and adults. For example, might gender influence (i.e., moderate) the relationships between personality traits, social reward, and engagement in trolling? And is psychopathy indirectly related to trolling via negative social potency?

Lastly, past research has shown that trolling behaviour in adults on social media is most common in posts involving politics or current events [30] and social media posts using highly opiniated words increased the chance of being trolled [80]. An audit of open social media accounts and qualitative analysis of trolling incidents (e.g., discourse analysis) could provide further insights into the sequence of behaviours and potential intervention points to accompany the knowledge garnered in this study.

## Conclusion

The current study provides the first investigation of Australian adolescent’s experiences of trolling and factors associated with this behaviour. We were particularly interested in exploring individual differences, such as personality traits and empathy, that have been implicated in adults who troll. We have demonstrated similarities between adolescent and adult samples—specifically, for both age cohorts, gender, high psychopathy, and high negative social potency emerge as predictors of trolling. Our findings also demonstrate differences that may be key to understanding trolling behaviours across age cohorts. For adolescents, when sadism, psychopathy, and negative social potency were included in the shared variance model, sadism was no longer a predictor of trolling—a finding not observed in an adult sample [58]. We discovered that when controlling for negative social potency, the relationship between sadism and trolling was no longer significant. It appears that both adults and adolescents troll for the enjoyment of antisocial interpersonal interactions (i.e., negative social potency), but the desire to cause harm (i.e., sadism) is less relevant for adolescents. These findings provide novel insight into the psychological profile of the adolescent who trolls, highlighting social interactions as a fundamental avenue for future investigation.

## Supporting information

S1 Table Participant demographics: Social media use and behaviour. (DOCX)

S2 Table Bivariate correlations by gender (boys and girls) for age, psychopathy, sadism, self-esteem, cognitive empathy, affective empathy, and negative social potency, and trolling. (DOCX)

S1 Appendix Data screening and assumption testing. (DOCX)

S2 Appendix Supplementary analyses using trolling frequency data. (DOCX)
