# Supplementary material for: An exploration of trolling behaviours in Australian adolescents: An online survey
Source: PLoS One. 2023 Apr 12;18(4):e0284378. doi: 10.1371/journal.pone.0284378 (PMC10096273; doi:10.1371/journal.pone.0284378)
Supplement: S1 Table — (DOCX) [file pone.0284378.s001.docx]

**S1 Table. Participant demographics: Social media use and behaviour.**

| **Time spent on social media per day** | **Percentage** |
| --- | --- |
| Under 30 minutes | 3.8% |
| 30 minutes – 1 hour | 14% |
| 1-2 hours | 26.1% |
| 3 hours | 31.2% |
| 4 - 5 hours | 19.1% |
| 6 – 8 hours | 3.2% |
| More than 8 hours | 2.5% |
| **Currently using social media platform** |  |
| Instagram | 77.1% |
| YouTube | 71.3% |
| Snapchat | 65.6% |
| Facebook | 59.2% |
| TikTok | 56.1% |
| Pinterest | 39.5% |
| WhatsApp | 31.8% |
| Twitter | 25.5% |
| Tumblr | 10.8% |
| Discord | 6.3% |
| WordPress | 5.7% |
| WeChat | 5.1% |
| **Purpose of social media use** |  |
| Keep in touch with family and friends | 84.7% |
| To share videos/pictures/music | 65% |
| To find information | 51.6% |
| To play games | 38.9% |
| To make new friends | 33.8% |
| To share your experience | 30.6% |
| To get opinions | 15.9% |
| **Access social media** |  |
| Smartphone | 90.4% |
| Laptop | 49% |
| PC | 20.4% |
| iPod/iPad | 20.4% |
| **Personal information included on social media** |  |
| Pictures of self | 63.1% |
| Real name | 62.4% |
| Interests | 45.9% |
| Hobbies | 37.6% |
| Email | 30.6% |
| Videos of self | 28.7% |
| Date of birth | 21.7% |
| Relationship status | 14.6% |
| Mobile number | 12.7% |
| Town/residence | 5.1% |
