# Supplementary material for: An exploration of trolling behaviours in Australian adolescents: An online survey
Source: PLoS One. 2023 Apr 12;18(4):e0284378. doi: 10.1371/journal.pone.0284378 (PMC10096273; doi:10.1371/journal.pone.0284378)
Supplement: S2 Table — (DOCX) [file pone.0284378.s002.docx]

**S4 Table. Bivariate correlations by gender (boys and girls) for age, psychopathy, sadism, self-esteem, cognitive empathy, affective empathy, and negative social potency, and trolling**

|  | 1. | 2. | 3. | 4. | 5. | 6. | 7. | 8. |
| --- | --- | --- | --- | --- | --- | --- | --- | --- |
| 1. Age | - | -.16_a_ | -.13_a_ | .11_a_ | -.11_a_ | .05_a_ | -.13_b_ | .12_a_ |
| 2. Psychopathy | .09_a_ | - | .52***_a_ | .06_a_ | -.08_a_ | -.37***_b_ | .56***_a_ | .24*_b_ |
| 3. Sadism | .04_a_ | .53***_a_ | - | -.03_a_ | .00_a_ | -.31**_b_ | .69***_a_ | .16_b_ |
| 4. Self-esteem | .03_a_ | .10_a_ | .21_a_ | - | .15_b_ | .12_b_ | -.00_a_ | -.02_a_ |
| 5. Cognitive empathy | -.01_a_ | -.13_a_ | -.08_a_ | -.17_a_ | - | .31**_a_ | -.14_a_ | -.12_a_ |
| 6. Affective empathy | -.01_a_ | .17_a_ | .06_a_ | -.32*_a_ | .23_a_ | - | -.28**_b_ | -.09_a_ |
| 7. Negative social potency | .21_a_ | .59***_a_ | .69***_a_ | .21_a_ | .08_a_ | .08_a_ | - | .33**_b_ |
| 8. Trolling | .10_a_ | .59***_a_ | .46***_a_ | .19_a_ | -.10_a_ | .08_a_ | .60***_a_ | - |

*Note.* **p* < .05, ***p* < .01, ****p* < .001; Correlations coefficients above diagonal line are for girls, correlations coefficients below diagonal line are for boys; different subscript indicates correlations between boys and girls differ at Fisher’s *z*, *p* < .05.
