# Supplementary material for: An exploration of trolling behaviours in Australian adolescents: An online survey
Source: PLoS One. 2023 Apr 12;18(4):e0284378. doi: 10.1371/journal.pone.0284378 (PMC10096273; doi:10.1371/journal.pone.0284378)
Supplement: S1 Appendix — (DOCX) [file pone.0284378.s003.docx]

**S2 Appendix**

**Data screening and assumption testing**

**Data screening for bots**

Data from Phases 1 and 2 were compiled, and initial screening indicated potential issues with bot generated responses (e.g., multiple responses with the same start and end times) [see Storozuk et al., 2020]. When examining data across multiple variables (e.g., start-time, end-time, free-text postcode response), it became apparent there were issues with the legitimacy of many responses. The primary indicators of bot generated data were large volumes of cases completed in short periods of time (e.g., 10 new participants all commencing at approximately the same time), with identical (or very similar) start and end times/dates, coupled with either identical location information (i.e., city/town/postcode; that was either correct e.g., Melbourne, or incorrect e.g., XYFVDPS) or similar stylistic patterns of location information. It is important to note that, while some of the variables alone (e.g., the time-based variables) provided a good indication of suspicious activity, other single variables (e.g., location) examined in isolation did not raise concerns. Rather, it was the combination of these indicators that resulted in the detection, and removal, of bot generated responses. The same screening process was undertaken for the data collected in Phase 3, with data collection also proactively monitored in this phase. That is, participants time-based and location responses were monitored throughout the period of data collection. The data was examined again in detail upon survey close.

In addition to the removal of suspected bot responses, a small number of participants were excluded from analyses for other reasons (e.g., rapid response time). Table 2 provides a detailed overview of data removed from each recruitment phase. It should be noted that simple statistical techniques have been suggested as effective in detecting bot activity (e.g., Mahalanobis distance), at least when responses are in a random pattern [Dupuis et al., 2019]. Mahalanobis distance was computed for all participants (including suspected bot cases), with only two cases identified as potentially problematic. These cases did not have other flags to suggest they were bot generated but were removed from the final sample (outlined in more detail as part of the assumption testing for the primary analyses).

**S3 Table. Overview of data cleaning across the three phases of recruitment.**

| **Phase** | **Complete** | **Excluded: Bot** | **Excluded: Other** | **Retained** |
| --- | --- | --- | --- | --- |
| 1 | 108 | 40 | 1 (outlier) | 67 |
| 2 | 126 | 79 | 1 (postcode) | 46 |
| 3 | 47 | 0* | 3 (1 outlier, 2 rapid response times) | 44 |
| **Total** | 279 | 119 | 5 | 157 |

*Note.* *Phase 3 employed the use of a CAPTCHA

**Data screening for main analyses**

Data were initially checked for missing values and no missing values were present. The standardised residuals scatterplot was assessed for assumptions of linearity and homoscedasticity, and assumptions were considered met. The standardised residuals histogram was assessed for normality, and the assumption was considered met. Distributions of each variable were assessed for univariate normality. All normality tests (i.e., Kolmogorov-Smirnov) were significant; however, as these tests are considered sensitive to minor violations of normality, standardised skew and kurtosis values were calculated and examined. Based on these standardised values, negative social potency, sadism, and trolling distributions deviated from the normal distribution. As these deviations were minor, consistent with previous findings, and as the *F* test is considered robust to minor violations of normality [Keppel & Wickens, 2004], we decided against transforming the distribution.

Univariate outliers were screened ±3.29 *SD* of the *M*. One univariate outlier was detected above the *M* on the negative social potency distribution. Multivariate outliers were assessed through generating Mahalanobis distance values and assessed with the critical value cut-off of 24.32 (*df* = 7). Two multivariate outliers were identified. The univariate outlier was assessed and determined to be a legitimate response and was retained for analyses. Both multivariate outliers were associated with inconsistent patterns of responses (e.g., strongly disagree on all items including reverse scored), and as such, were removed from data for analyses.

One-way ANOVAs, corrected for alpha inflations, were run for all predictor variables (psychopathy, sadism, self-esteem, cognitive empathy, affective empathy, negative social potency) and the criterion variable (trolling [GAIT]) comparing participants across Phase 1, Phase 2, and Phase 3 of recruitment. There were no significant differences between groups on the aforementioned variables (all *p*s > .05) excluding self-esteem, *F*(2, 154) = 3.26, *p* = 0.041. A Bonferroni adjusted post hoc test revealed self-esteem scores were statistically higher for participants recruited at Phase 3 (*M* = 25.36, *SD* = 6.43) compared to those recruited at Phase 2 (*M* = 22.52, *SD* = 5.84). There were no statistically significant differences between the self-esteem scores of participants recruited at Phases 1 (*M* = 23.24, *SD* = 4.59) and 2, or between Phases 1 and 3.

**References**

Dupuis M, Meier E, Cuneo F. Detecting computer-generated random responding in questionnaire-based data: A comparison of seven indices. Behavior Research Methods. 2019; 51:2228–37. doi:10.3758/s13428-018-1103-y

Keppel G, Wickens TD. Design and analysis: A researchers handbook. 4 ed: Pearson; 2004.

Storozuk A, Ashley M, Delage V, Maloney EA. Got Bots? Practical Recommendations to Protect Online Survey Data from Bot Attacks. The Quantitative Methods for Psychology. 2020;16(5):472-81. doi:10.20982/tqmp.16.5.p472.
