# Supplementary material for: An exploration of trolling behaviours in Australian adolescents: An online survey
Source: PLoS One. 2023 Apr 12;18(4):e0284378. doi: 10.1371/journal.pone.0284378 (PMC10096273; doi:10.1371/journal.pone.0284378)
Supplement: S2 Appendix — (DOCX) [file pone.0284378.s004.docx]

**S3 Appendix**

**Supplementary analyses using trolling frequency data**

To further explore the relationship between being trolled and trolling others (“No” response coded as 0 and “Yes” response coded as 1), a bivariate correlation was computed, *r*(157) = .30, *p* < .001, indicating that individuals who had experienced trolling, were more likely to troll others and those who had not experienced trolling, were less likely to troll others. To further explore potential differences between experiences of trolling and social media use, a series of independent samples *t*-tests were run. The first test showed no difference in hours spent on social media per day between adolescents who had been trolled (*M* = 4.68, *SD* = 1.23) and adolescents who had not been trolled (*M* = 4.66, *SD* = 1.31), *t*(155) = -0.08 *p* = .933. The second test also found no difference in hours spent on social media per day between adolescents who had trolled others (*M* = 4.57, *SD* = 1.12) and adolescents who had not trolled others (*M* = 4.68, *SD* = 1.32), *t*(155) = 0.37, *p* = .711. There was also no difference in the total number of social media sites used between adolescents that had been trolled (*M* = 4.81, *SD* = 2.06) and had not been trolled (*M* = 4.51, *SD* = 1.84), *t*(155) = -0.86, *p* = .394, and no difference between adolescents that had trolled others (*M* = 4.47, *SD* = 2.07) and had not trolled others (*M* = 4.60, *SD* = 1.88), *t*(155) = 0.28, *p* = .777.
